# Circulating Plasma MiR-141 Is a Novel Biomarker for Metastatic Colon Cancer and Predicts Poor Prognosis

**DOI:** 10.1371/journal.pone.0017745

**Published:** 2011-03-17

**Authors:** Hanyin Cheng, Lina Zhang, David E. Cogdell, Hong Zheng, Aaron J. Schetter, Matti Nykter, Curtis C. Harris, Kexin Chen, Stanley R. Hamilton, Wei Zhang

**Affiliations:** 1 Department of Pathology, The University of Texas M. D. Anderson Cancer Center, Houston, Texas, United States of America; 2 Department of Epidemiology and Biostatistics, Tianjin Medical University Cancer Institute and Hospital, Tianjin, China; 3 Laboratory of Human Carcinogenesis, Center for Cancer Research, National Cancer Institute, Bethesda, Maryland, United States of America; 4 Department of Signal Processing, Tampere University of Technology, Tampere, Finland; University of Barcelona, Spain

## Abstract

**Background:**

Colorectal cancer (CRC) remains one of the major cancer types and cancer related death worldwide. Sensitive, non-invasive biomarkers that can facilitate disease detection, staging and prediction of therapeutic outcome are highly desirable to improve survival rate and help to determine optimized treatment for CRC. The small non-coding RNAs, microRNAs (miRNAs), have recently been identified as critical regulators for various diseases including cancer and may represent a novel class of cancer biomarkers. The purpose of this study was to identify and validate circulating microRNAs in human plasma for use as such biomarkers in colon cancer.

**Methodology/Principal Findings:**

By using quantitative reverse transcription-polymerase chain reaction, we found that circulating miR-141 was significantly associated with stage IV colon cancer in a cohort of 102 plasma samples. Receiver operating characteristic (ROC) analysis was used to evaluate the sensitivity and specificity of candidate plasma microRNA markers. We observed that combination of miR-141 and carcinoembryonic antigen (CEA), a widely used marker for CRC, further improved the accuracy of detection. These findings were validated in an independent cohort of 156 plasma samples collected at Tianjin, China. Furthermore, our analysis showed that high levels of plasma miR-141 predicted poor survival in both cohorts and that miR-141 was an independent prognostic factor for advanced colon cancer.

**Conclusions/Significance:**

We propose that plasma miR-141 may represent a novel biomarker that complements CEA in detecting colon cancer with distant metastasis and that high levels of miR-141 in plasma were associated with poor prognosis.

## Introduction

Colorectal cancer (CRC) is a worldwide health problem with 655,000 deaths per year [1]. In the United States, CRC is the third most common cancer type and the second most common cause of cancer-related death [2], with an estimated 51,370 deaths in 2010 according to the National Cancer Institute. In China, CRC remains the fifth most common cancer type and the fourth most common cause of cancer-related death [3]. Despite early screening and development of new chemotherapeutic strategies, CRC survival rates during the past 20 years have not substantially improved. Moreover, the incidence of CRC is increasing rapidly in recent years in China [4]. Novel biomarkers that are of clinical value are thus in urgent need to improve compliance rates. Carcinoembryonic antigen (CEA) has been used as a serum marker of CRC, although its sensitivity has varied in different studies [5], [6]. Recently, a family of small regulatory RNAs, microRNAs, has emerged as possible plasma markers for human diseases including cancers due to their relative stability in the circulation [7].

MicroRNAs are small non-coding RNAs (18–22 nt in length) that regulate the expression of target genes by interfering with transcription or inhibiting translation [8]. Studies have demonstrated that microRNAs play a crucial role in almost all cellular biological processes including metabolism, survival, differentiation and apoptosis. Deregulation of specific microRNAs contributes to a variety of diseases, most notably the development and progression of cancer, including CRC. Specifically, microRNA expression profiling in CRC showed that a number of microRNAs, including miR-21, miR-20a and miR155 were upregulated in tumor tissues and that higher miR-21 was associated with poor therapeutic outcome [9], [10]. The potential of circulating miRNAs in plasma as cancer biomarkers has also been evaluated in a few studies. For CRC, a recent study reported that miR-92 levels were significantly higher in plasma samples from patients than in healthy controls and can be a potential marker for CRC detection [11]. A study of plasma samples from prostate cancer patients reported that plasma miR-141 levels can be used to screen for metastatic prostate cancer with high sensitivity [12].

In this proof-of-principle study to identify potential biomarkers for CRC, we examined whether selected candidate microRNAs could serve as non-invasive, blood-based markers for CRC by analyzing the relative levels of three microRNAs (miR-21, miR-92, and miR-141) in a cohort of 102 plasma samples from healthy individuals and CRC patients obtained from TexGen, a collaboration of Texas Medical Center Institutions. Our initial data indicated that among these three microRNAs, plasma miR-141 levels were significantly elevated in the plasma of colon cancer patients with Stage IV disease and could readily discriminate distant metastasis cases from normal controls and patients with other stages. Combination of miR-141 with CEA was complementary and could further increase the detection accuracy of distant metastasis in colon cancer. These findings were validated in an independent cohort of 156 plasma samples obtained from Tianjin, China. Our further analyses provided supporting evidence that plasma miR-141 was a potential prognostic factor that predicted for poor survival in colon cancer patients. Interestingly, unlike in plasma, miR-141 was not differentially expressed in tumor tissues between Stage IV and Stage I–II colon cancer patients or between tumor tissues and adjacent non-tumor tissues in Stage IV patients, suggesting that elevation of plasma miR-141 in Stage IV patients might be derived from other systemic responses such as inflammatory reactions in these patients.

## Materials and Methods

### Ethics statement

Both plasma- and tissue-based studies were approved by the Institutional Review Board (IRB) at the MD Anderson Cancer Center and by the Ethics Committee at the Tianjin Medical University Cancer Institute and Hospital. All participants gave written consent of their information to be stored in the hospital database and used for research.

### Clinical samples

Two independent sets of plasma samples were used. A total of 102 plasma samples from age- and gender- matched healthy individuals and CRC patients (Stage I–IV) were obtained from TexGen between 2002 and 2008, a collaboration of Texas Medical Center institutions that provides biological samples as well as epidemiological and clinical data (TexGen samples, See Table S1). An independent cohort of 156 plasma samples from age- and gender- matched healthy donors and colon cancer patients (Stage I–IV) were obtained at the Tianjin Medical University Cancer Institute and Hospital, Tianjin, China (TCH samples) between 2007 and 2009 (See Table S2). Pathologic classification of disease in all patients was performed following the International Union Against Cancer (UICC) and American Joint Committee on Cancer (AJCC) TNM staging system for colon cancer established in 2003. Blood samples were collected from all patients before operation and therapy. CEA levels of both TexGen and TCH plasma samples were measured by standard enzyme immunoassay as part of routine clinical tests and were acquired from the clinical database at each institute. Follow-up data of all the recruited colon cancer patients from both TexGen and TCH were acquired and survival time was calculated from date of diagnosis to the date of death or last follow-up in June, 2010. All patients recruited to this study had not received chemotherapy or radiotherapy prior to the blood draws. The demographic information of the two sets of samples is summarized in the supplemental material.

For the tissue-based analysis tumor tissues from 21 colon cancer patients with distant metastatic disease (Stage IV) and 24 colon cancer patients with non-metastatic disease (Stage I and II) were collected between 2007 and 2009 by Tianjin Medical University Cancer Institute and Hospital from the same patients whose plasma samples were used.

### RNA isolation and quantitative RT-PCR

Small RNA was enriched from all plasma samples using the mirVana PARIS RNA isolation kit (Ambion, Austin, TX). Briefly, 250 µL of plasma was thawed on ice and centrifuged at 14,000 rpm for 10 minutes to remove cell debris and other cellular organelles. Next, 150 µL of supernatant was lysed with an equal volume of 2x denaturing solution. For normalization of sample-to-sample variation during the RNA isolation procedures, 25 fmol of synthetic *C. elegans* miRNA cel-miR-39 was added to each denatured sample. Small RNAs were then enriched and purified following manufacturer's protocol, with the exception that the enriched small RNAs were eluted in 45 µL of preheated nuclease-free water. Standard TRIZOL method (Invitrogen) was used to isolate total RNA from colon tissues.

For microRNA based RT-PCR assays, 2.5 µL of enriched small RNAs from plasma samples were reverse transcribed using the TaqMan MicroRNA Reverse Transciption Kit (Applied Biosystems, San Diego, CA) according to manufacturer's instructions in a total reaction volume of 7.5 µL. A 1∶20 dilution of RT products was used as template for the PCR stage. PCR reaction was performed in triplicates using TaqMan 2x Universal PCR Master Mix with conditions as described previously [13]. No-template controls for both RT step and PCR step were included to ensure target specific amplification. The 7900 Sequence Detection System 2.3 (Applied Biosystems) software defaults were used to compute the relative change in RNA expression by the 2^−ΔΔCt^ method with 95% confidence intervals. Assays for the TexGen specimens were performed at MD Anderson and assays for the TCH specimens were performed at Tianjin Medical University Cancer Institute and Hospital.

### MicroRNA profiling

MicroRNA microarray profiling was performed with RNA isolated from the Maryland cohort using the Ohio State microRNA microarray version 2.0 as previously described [9]. Microarray data (including raw and processed data) have been deposited in National Center for Biotechnology Information's (NCBI's) Gene Expression Omnibus (NCBIGEO GSE7828).

### Statistical analysis

The statistical significance was determined by using the Wilcoxon signed-rank tests between groups. Receiver operating characteristic (ROC) curves were generated to assess the diagnostic accuracy of each parameter, and the sensitivity and specificity of the optimum cut-off point were defined as those values that maximized the area under the ROC curve (AUC). The relative levels of microRNA were quantified using the 2^−ΔΔCt^ method, and the data were analyzed as the log_10_ of the relative quantity of the target microRNA. The statistical analysis was performed with the use of software packages SPSS version 16.0 (WPSS Ltd., Surrey, United Kingdom) and graphs were generated using Graphpad Prism 5.0 (Graphpad Software Inc, California). Spearman Correlation analysis was performed to reveal correlation between plasma miR-141 expression and CRC stages. All statistical tests were two-sided, and a *P* value of 0.05 was considered significant. The correlation between overall survival and plasma miR-141 was analyzed using Kaplan-Meier method and Log-rank test. Cox proportional-hazards regression analysis was used to evaluate whether plasma miR-141 was an independent prognostic factor for colon cancer.

## Results

### Plasma miR-141 levels are correlated with clinical stages in colon cancer

**Figure 1:**
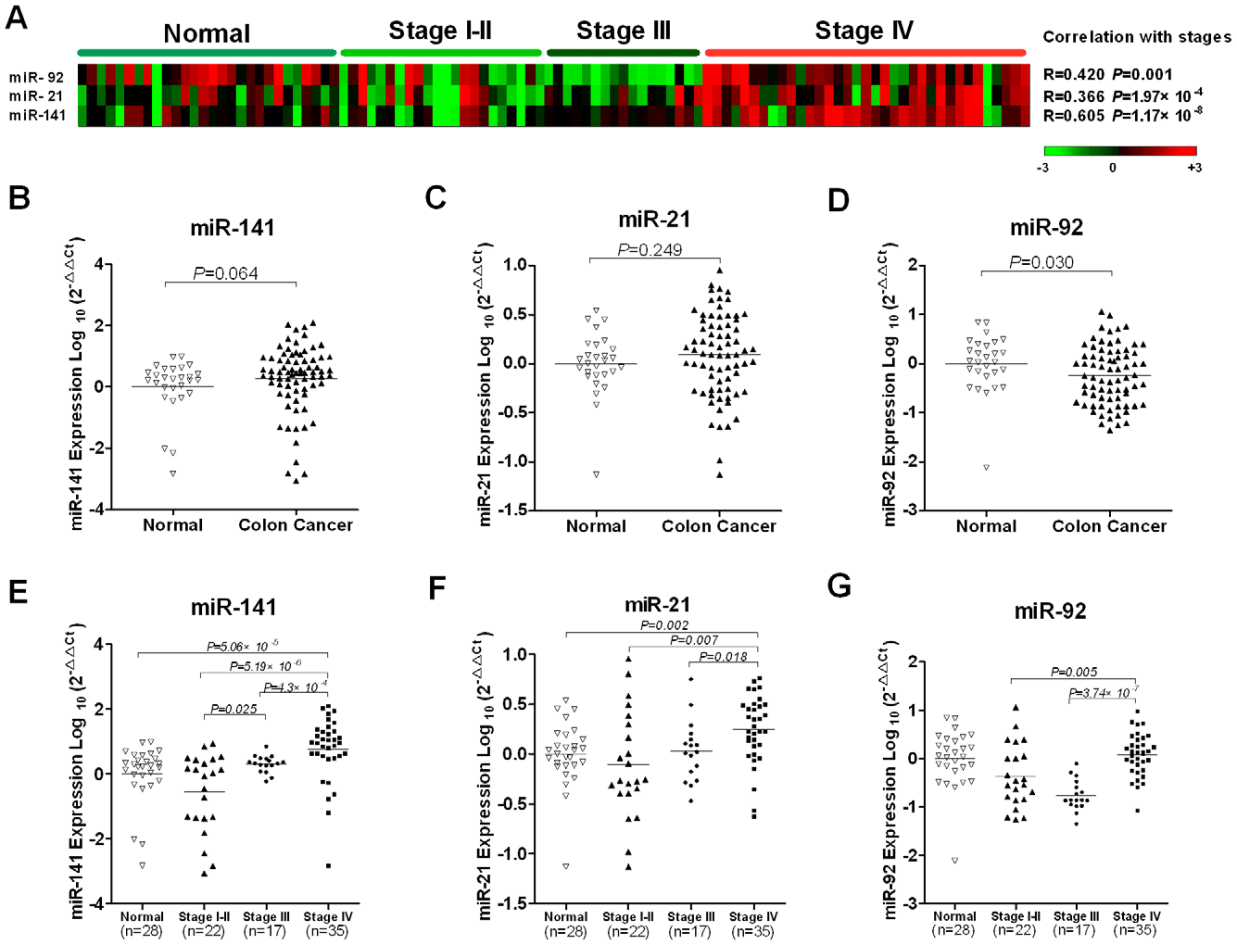
Plasma miR-141, miR-21 and miR-92 levels in healthy controls and colon cancer patients. (A) Each row corresponds to a plasma miRNA and each column corresponds to each sample. Expression levels for each miRNA are normalized across the samples and shaded in colors such that red denotes high expression and green denotes low expression. Spearman correlation shows that miR-141 is highly correlated with stages (r = 0.605, P = 1.17×10^−8^). (B–G) The relative levels of selected plasma microRNAs were normalized to spike-in control cel-miR-39 and shown as the log10 of the relative quantity (RQ). The Wilcoxon two-sample tests were performed to examine the difference of selected plasma microRNAs between normal controls and colon cancer patients (B–D), or between normal controls and/or colon cancer patients with different clinical stages (E–G).

Three microRNAs (miR-21, miR-92 and miR-141) were selected in our studies to examine their potential to serve as biomarkers for colon cancer. These candidate miRNAs were selected because 1) miR-21 has been found to be upregulated in CRC tumor tissues [9], [10], [14]; 2) miR-92 has recently been reported to be a plasma marker for colon cancer [11]; and 3) miR-141 has been shown to be a potential plasma marker for metastatic prostate cancer [12]. Quantitative RT-PCR based microRNA assays on these three miRNAs were performed. We first examined the correlation between these three miRNAs and colon cancer clinical stages. Stage I and Stage II cases were grouped together in our analysis because only limited Stage I cases were available. Heat-map analysis showed that the miR-141 levels clustered according to different stages (Figure 1, A). Spearman correlation analysis showed that the plasma miR-141 levels were highly correlated with colon cancer stages (r = 0.605, P = 1.17×10^−8^). In contrast, miR-21 and miR-92 were less correlated with clinical stages (Figure 1, A).

We next analyzed whether these candidate microRNAs could serve as circulating colon cancer markers by comparing their plasma levels between cancer patients and normal controls. Surprisingly, unlike the previous report that upregulation of plasma miR-92 was a colon cancer biomarker, the Wilcoxon two-sample test showed that among these three microRNAs, plasma miR-92 was significantly decreased in the TexGen cohort of colon cancer patients (*p* = 0.03) (Figure 1, D). However, the decrease in plasma miR-92 was only observed in Stage I–II and Stage III and the level increased to a similar level as that of normal controls in Stage IV colon cancer (Figure 1, G). Plasma levels of miR-21 and miR-141 did not show significant difference between controls and cancer patients (Figure 1, B & C). After stratification of the cancer patients according to their clinical stages, plasma miR-141 was significantly upregulated in Stage IV colon cancer (Figure 1, E). To a much less extent, plasma miR-21 was also shown to be elevated in Stage IV colon cancer (Figure 1, F). Based on these observations, we sought to focus on miR-141 for further characterization.

### High plasma miR-141 levels are associated with Stage IV colon cancer and complement with CEA in diagnosis

**Figure 2:**
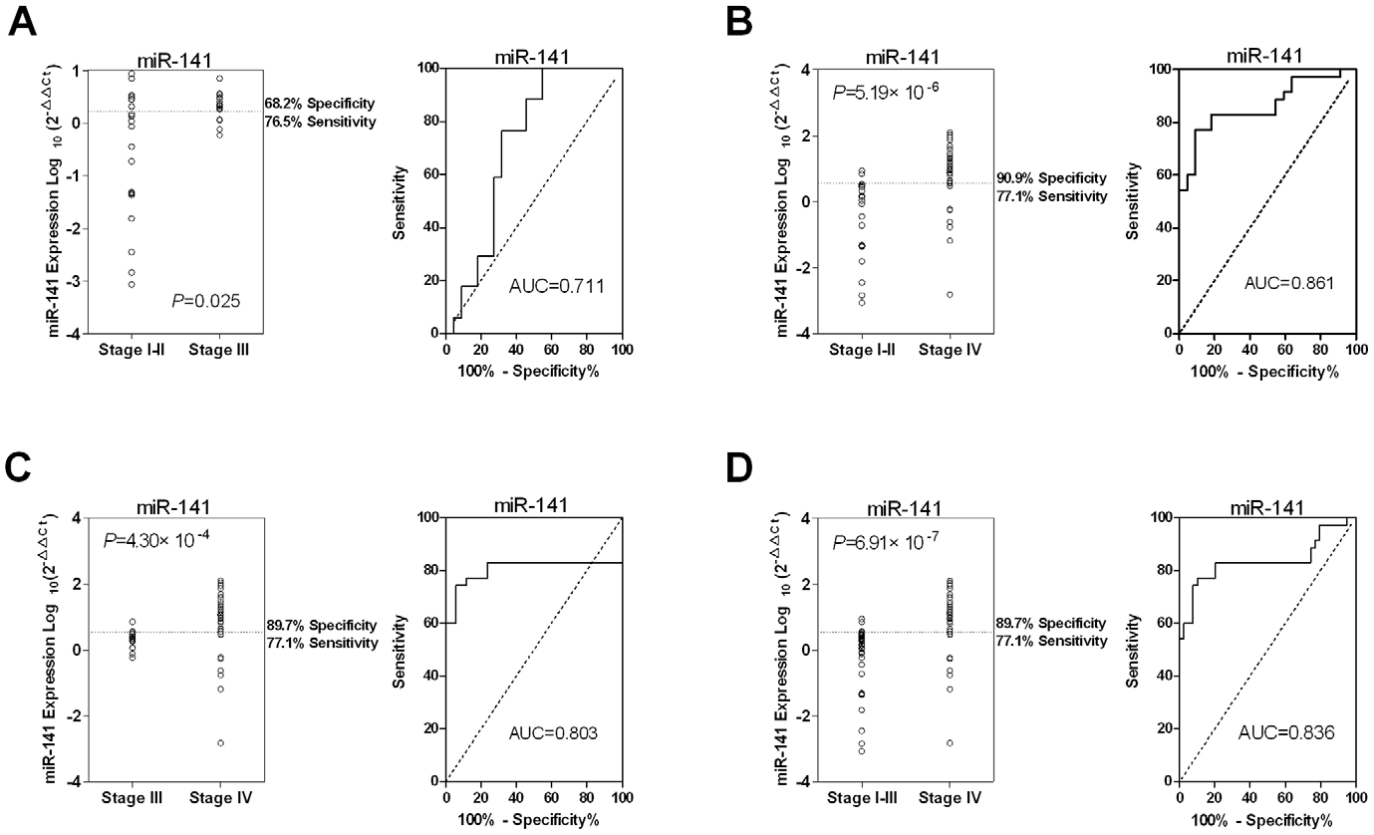
Higher plasma miR-141 is significantly associated with Stage IV colon cancer in the training set. (A) Small RNA was isolated from plasma samples and miR-141 was measured by quantitative RT-PCR assays. The Wilcoxon two-sample test was performed to evaluate differences of miR-141 levels between the Stage III and Stage I–II groups. ROC analysis was performed to determine the sensitivity and specificity with the value of AUC in the right panel. The Wilcoxon two-sample tests between (B) Stage IV and Stage I–II, (C) Stage IV and Sage III, and (D) Stage IV and Stage I–III groups were performed to evaluate the association of plasma miR-141 with Stage IV disease status.

The above results demonstrated that among the three selected microRNAs, miR-141 was significantly correlated with colon cancer stages. The detailed Wilcoxon two-sample test showed that the plasma miR-141 was significantly elevated in Stage IV cases when compared with Stage I–II, Stage III and Stage I–III combined. The ROC curve was plotted to identify a cut-off value that could distinguish stage IV colon cancer from other groups. ROC curve analysis showed that at the optimal cut-off, plasma miR-141 had a 90.9% sensitivity and a 77.1% specificity in separating Stage IV cases from Stage I–II cases with an AUC of 0.861 (Figure 2, B), a 77.1% sensitivity and a 89.7% specificity in separating Stage IV and Stage III cases with an AUC of 0.803 (Figure 2, C), and a 77.1% sensitivity and a 89.7% specificity in separating the Stage IV and combined Stage I–III cases with an AUC of 0.836 (Figure 2, D). Our analysis also revealed that in this cohort, plasma miR-141 was significantly elevated in Stage III patients compared with early stage patients (*p* = 0.025) (Figure 2, A). These results suggest that circulating miR-141 might be a novel biomarker for metastatic colon cancer.

**Figure 3:**
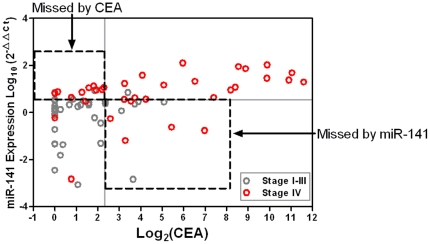
Combination of CEA and miR-141 identifies additional Stage IV cases in the training cohort. Two-parameter (expression of miR-141 and CEA in plasma) classification is used to discriminate distant metastatic colon cancer. The cut-off value for CEA is 5.0 ng/mL, and for miR-141 is 16.77 defined from the ROC curve. The corresponding cut-off values are marked by grey lines.

Because the blood CEA test is widely used marker for CRC patients, we sought to compare the performance of miR-141 with CEA as a biomarker. We examined whether a combination of miR-141 and CEA was more sensitive than either marker when used individually. Our analysis showed that they were indeed complementary. When the specificity was set at 100%, miR-141 identified seven Stage IV colon cancer that were missed by CEA alone and CEA identified four Stage IV metastatic colon cancer cases that were missed by miR-141 alone (Figure 3).

### Validation of plasma miR-141 as a colon cancer marker in an independent population

Results from the above studies provided evidence that high levels of circulating miR-141 could be a potential biomarker that complements CEA for metastatic colon cancer. To further validate the potential utility of miR-141 in the clinical management of colon cancer, we performed a validation study with an independent set of plasma samples (TCH samples) (See Table S2 for demographic information of TCH cases). The measurements were made independently following the same experimental procedures used for the TexGen samples.

**Figure 4:**
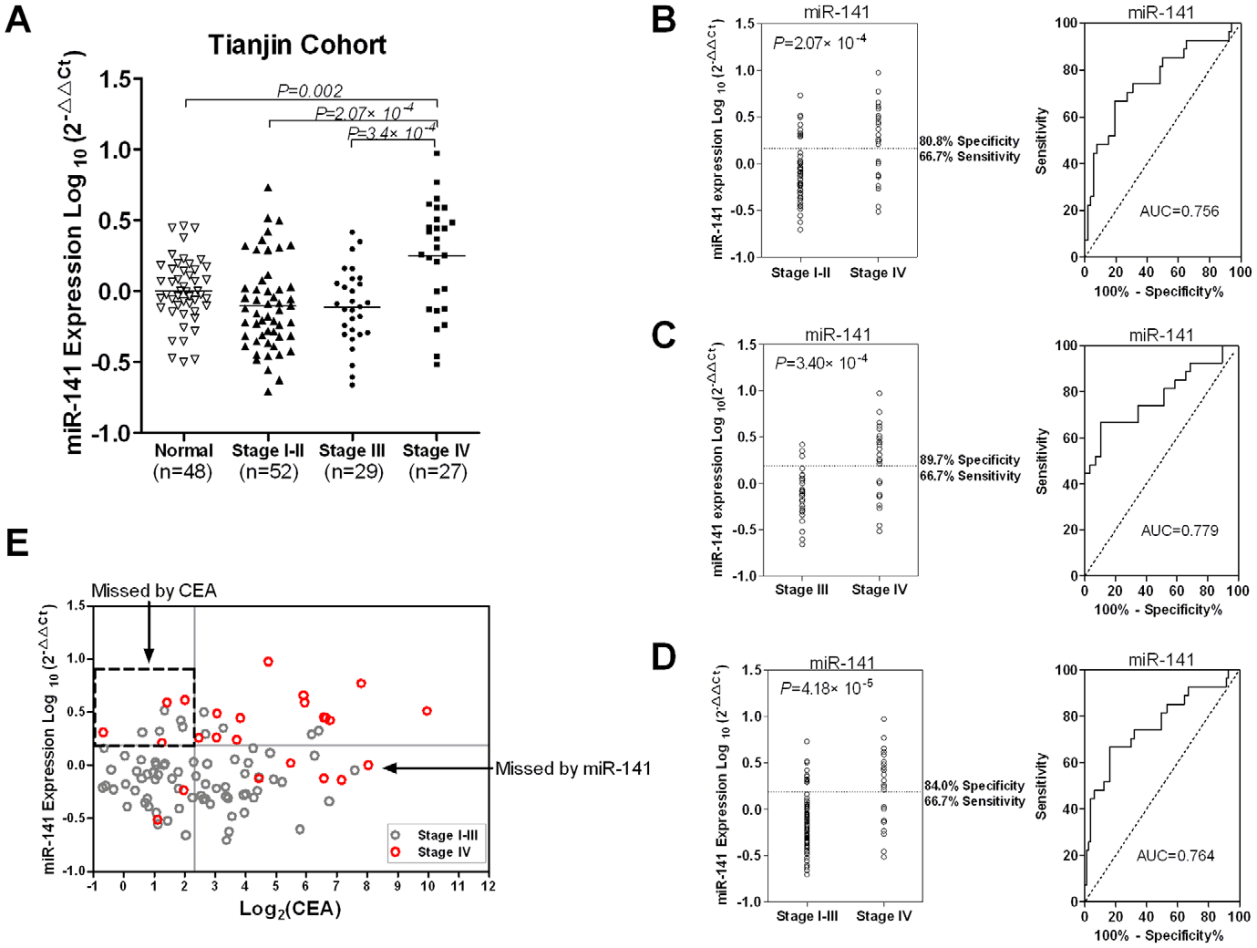
Higher plasma miR-141 level is associated with Stage IV colon cancer patients in the validation data set. Small RNA isolation and miR-141 quantitative RT-PCR assays were performed in the same way as for the training cohort. (A) The Wilcoxon two-sample test was performed to compare miR-141 levels between normal controls and/or colon cancer patients with different clinical stages. (B–D) The same analyses were performed to compare miR-141 levels between Stage IV cases and Stage I–II cases (B), between Stage IV and Stage III cases (C), and between Stage IV and Stage I–III cases (D), respectively. ROC analysis was performed to determine the sensitivity and specificity with the value of AUC in the right panel. (E) Combination of CEA and miR-141 identified additional metastatic patients that were missed by either marker alone. See Figure 3 for the definition of the cut-off values for CEA and miR-141.

The results validated that the plasma miR-141 level was significantly higher in Stage IV patients than in normal controls, Stage I–II and Stage III patients (Figure 4, A). Specifically, the ROC curves showed that a cut-off for plasma miR-141 could be determined with a 66.7% sensitivity and a 80.8% specificity for separating the Stage IV cases from the Stage I–II cases (AUC = 0.756) (Figure 4, B), a 66.7% sensitivity and a 89.7% specificity for separating from Stage III cases (AUC = 0.779) (Figure 4, C), and a 66.7% sensitivity and a 84.0% specificity for separating from Stage 1–III cases (AUC = 0.764) (Figure 4, D). Unlike in TexGen plasma samples, we however failed to detect a significant difference in miR-141 levels between Stage III and Stage I–II cases (not shown), suggesting that higher plasma miR-141 is more associated with distant and less with lymph-node metastasis in colon cancer.

We also evaluated the CEA data in the TCH dataset. Similar to what was shown in the TexGen samples, combination of miR-141 and CEA identified additional metastatic cases that were otherwise missed by either marker used alone (Figure 4, E). This finding validated that the combination of these two biomarkers was a more effective approach for detecting Stage IV CRC patients.

### Plasma miR-141 is correlated with poor survival in colon cancer

To further evaluate whether plasma miR-141 levels can predict prognosis, we next performed a survival analysis on TexGen and TCH cases. Kaplan-Meier survival curves showed that higher expression of plasma miR-141 was significantly correlated with poor survival both in TexGen (*P* = 0.004, log-rank test) and Tianjin (*P* = 0.002, log-rank test) cohorts (Figure S1, A and B). Univariate Cox regression analysis demonstrated that plasma miR-141 was a significant prognostic indicator of the colon cancer in TexGen (HR = 3.80, 95%CI = 1.46−91) and Tianjin cases (HR = 4.83, 95%CI = 2.06−11.35), respectively (Table 1). To avoid any potential bias between the TexGen cohort and Tianjin cohort, we performed the univariate and multivariate survival analyses for all cases. The multivariate Cox proportional hazard regression analysis showed that plasma miR-141was an independent prognostic marker in colon cancer patients when we merged the samples from both centers (HR = 2.40, 95%CI = 1.18−4.86) (Table 1).

**Table 1:** Plasma miR-141 is an independent prognostic factor by Cox regression analysis.

	Univariate analysis		Multivariate analysis	
	Hazard ratio (95% CI)	*P*_1_	Hazard ratio (95% CI)	*P*_2_
TexGen				
miR-141	3.80(1.46, 9.91)	0.006	1.36(0.45, 4.14)	0.589
Sex	0.43(0.20, 0.97)	0.042	0.45(0.19, 1.05)	0.063
Age	1.40(0.97, 1.12)	0.280	1.03(0.97, 1.10)	0.364
Stage	3.56(1.79, 7.09)	3.07×10^−4^	3.37(1.50, 7.55)	0.003
Tianjin				
miR-141	4.83(2.06, 11.35)	2.98×10^−4^	3.41(1.36, 8.56)	0.009
Sex	1.47(0.63, 3.41)	0.370	1.02(0.43, 2.44)	0.964
Age	0.99(0.96, 1.03)	0.710	0.99(0.97, 1.03)	0.578
Stage	3.82(2.11, 6.93)	1.05×10^−5^	3.22(1.75, 5.92)	1.67×10^−4^
All Cases				
miR-141	3.61(1.96, 6.65)	3.80×10^−5^	2.40(1.18, 4.86)	0.016*
Sex	0.75(0.42, 1.35)	0.337	0.65(0.36, 1.19)	0.162*
Age	1.00(0.97, 1.04)	0.828	1.00(0.97, 1.03)	0.912*
Stage	3.30(2.14, 5.09)	6.55×10^−8^	3.16(1.97, 5.07)	1.90×10^−6^ *

*Adjusted by the different centers.

### MiR-141 is not differentially expressed in colon cancer tissues

**Figure 5:**
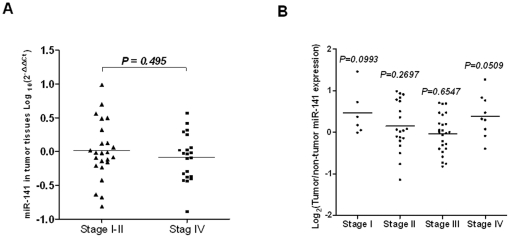
MiR-141 in tumor tissues is not associated with colon cancer tumor stage. (A) Total RNA was isolated from tumor tissues of metastatic or non-metastatic patients from the Tianjin cohort, and the miR-141 levels were measured with quantitative RT-PCR using RNU6B as an endogenous control. The Wilcoxon two-sample test was performed to compare miR-141 expression in tumor tissues between these two groups. A *P*<0.05 is considered significant. (B) The miR-141 expression levels were measured using microRNA profiling data from the Maryland cohort of CRC samples. The Wilcoxon matched-pairs tests were performed to compare the miR-141 levels between tumor tissues and adjacent normal tissues in these CRC patients.

We sought to determine whether the elevated miR-141 in plasma from Stage IV CRC patients reflected differential expression in the tumor tissues from different disease stages. We first compared miR-141 levels in tumor tissues between Stage I–II and Stage IV in the TCH cohort, which surprisingly showed that miR-141 was not upregulated in Stage IV cases (*p* = 0.495) (Figure 5, A). We next analyzed our previously described microRNA microarray profiling data (GEO, GSE7828). Our analysis also confirmed that the miR-141 level was not significantly higher in tumor tissues than in adjacent normal tissues in stage IV patients, as well as in patients of other stages (Figure 5, B).

## Discussion

In this study, we took a focused approach and examined three candidate microRNAs for their potential value as plasma biomarkers for colon cancer. Results from the Texas study revealed that plasma miR-141 was a sensitive marker and complemented CEA for detecting Stage IV colon cancer. This result was independently validated using a cohort of samples from colon cancer patients in Tianjin, China. Thus, this collaborative investigation carried out by two independent teams of researchers at two different sites on different ethnic patient populations provides strong evidence that miR-141 is a valid plasma marker that complements CEA in determining stage IV colon cancer. Importantly, our data also demonstrated that higher plasma miR-141 was associated with shorter survival in both cohorts and that miR-141 was an independent prognostic indicator for colon cancer. We believe that the identification of miR-141 may represent a key advancement in the search for valuable plasma markers for colon cancer that have the potential to be translated into clinical applications including prognosis, monitoring response to therapy, and detecting disease recurrence. Further validation in a larger cohort of samples and a prospective study will be needed to determine conclusively whether miR-141 serves as a circulating marker for late stage colon cancer.

Apart from the potential impact on clinical diagnosis and prognosis, this study also revealed some intriguing aspects regarding the origin of serum miRNAs in cancer. Results from this study and the previous study reporting miR-141 elevation in metastatic prostate cancer [12] link miR-141 to metastasis, we thus tested a straightforward hypothesis that the elevated plasma miR-141 reflected the elevated miR-141 level in tissue in Stage IV colon cancer patients. However surprisingly, we did not observe a significant difference in the miR-141 expression levels between tumor tissues and adjacent normal tissues among different stages of CRC or in tumor tissues between non-metastatic Stage I–II and metastatic Stage IV. Therefore, the elevation of miR-141 in plasma is not a simple indication of elevated miR-141 in corresponding tumor tissues at the primary site. It is possible that miR-141 is only elevated in the metastases at the secondary site. MiR-141 belongs to the miR-200 family, which promotes the mesenchymal-to-epithelial transition (MET) by inhibiting the expression of the E-cadherin transcriptional suppressors ZEB1 and ZEB2 [15], [16]. Thus it is consistent that we did not observe elevated miR-141 at the primary site of Stage IV colon cancer where EMT instead of MET is believed to occur resulting in cells with higher potential for cell migration and metastasis. When mesenchymal-like metastatic cells extravosate at distant site, a transition to epithelial (MET) occurs, which may explain the surge of miR-141 in metastatic cancer. This hypothesis will need to be tested with matching primary tumors and metastases in the future.

Another possibility is that selected cellular miRNAs contribute more significantly to the circulating miRNAs in cancer patients. The *in vitro* studies also showed that miRNA expression profiles in conditioned medium were different from those in cells, and those secreted miRNAs may represent a class of signaling molecules in mediating intercellular communication [17], although the mechanisms control microRNA packaging and secretion are largely unknown. In cancer patients especially at late stage, there are many systemic changes including inflammatory response that can involve immune systems and other organs. It is conceivable that these systemic responses may be a source of change in circulating miRNAs in cancer patients. Along this line, in addition to colon and prostate cancers, circulating miR-141 level has also been associated with other pathophysiological conditions including ovarian cancer [18] and pregnancy [19]. The difference in miR-141 in plasma between Stage IV and Stage I–II CRC may be related to differential inflammatory response in CRC and this possibility will need to be further investigated in the future.

Among our candidate miRNAs, miR-21 is an oncogene that is altered in many tumors by regulating the expression of multiple cancer-related target genes such as PTEN and TPM1 [20], [21]. Previous studies showed that expression of miR-21 was upregulated in CRC tumor tissues and was gradually elevated during tumor progression from early stage to late stage as compared with matched non-tumor tissues, suggesting that it would be a good circulating marker for CRC detection if this same elevation trend were seen in plasma. However, our analysis did not reveal a difference of plasma miR-21 between cancer patients and healthy controls. Although circulating miR-21 was also significantly elevated in patients with distant metastasis, the sensitivity and specificity were much lower than those for miR-141. Similarly, miR-92, a previously identified plasma markers for CRC in the Hong Kong cohorts did not represent a useful marker in our initial analysis of the TexGen cohort. We reason that the genetic variations among different ethic groups as well as environmental factors and diets may contribute to these conflicting conclusions.

Although the origin of the plasma miR-141 is yet to be resolved, our work suggested that the ongoing search for plasma miRNA markers can be a fruitful process. In comparison to other nucleotide molecules such as DNA and mRNA, miRNAs are resistant to DNase or RNase activity and, thus, are relatively stable in the circulation. In addition to our report here, there are increasing examples of plasma miRNAs as potential biomarkers. In acute leukemia patients, the plasma miR-92 level is dramatically reduced (similar to what we observed with our CRC in this study), and the ratio of miR-92a/miR-638 in plasma is a potential biomarker for this disease [22]. In addition, plasma levels of a panel of four miRNAs (miR-21, miR-210, miR-155, and miR-196a) were found to be potential markers for pancreatic cancer [23], and miR-31 upregulation in plasma may be a biomarker for oral cancer [24]. These studies showed the potential of using circulating miRNAs as biomarkers, but they are of limited value at present because of a lack of independent validations. In our study, the fact that plasma miR-141 was shown to be a prognostic factor in two independent cohorts consisting of two different ethnic populations provides compelling evidence that miR-141 may emerges as a valuable marker of clinical significance.

Although biomarkers for advanced cancer can be potentially used to monitor and predict therapeutic outcome, biomarkers that can detect early stage disease and monitor early metastasis are expected to represent more clinically relevant endpoints in the increase of overall survival rate. Future efforts are still needed to identify circulating microRNAs as biomarkers that can accurately detect CRC at its early stage.

## Supporting Information

Figure S1 **Higher miR-141 predicts poor prognosis in both cohorts.** Kaplan-Meier survival curves for colon cancer patients in both cohorts. The survival data were compared using the log-rank test and miR-141 expression levels in patients defined as high or low relative to the median. P-value of log-rank test is 0.004 and 0.002 in TexGen (A) and Tianjin (B) cohorts, respectively. Higher plasma levels of miR-141 were associated with poor overall survival in colon cancer patients.
(JPG)

Table S1 (DOC)

Table S2 (DOC)
