# Supplementary figures and images for: Circulating Plasma MiR-141 Is a Novel Biomarker for Metastatic Colon Cancer and Predicts Poor Prognosis
Source: PLoS One. 2011 Mar 17;6(3):e17745. doi: 10.1371/journal.pone.0017745 (PMC3060165; doi:10.1371/journal.pone.0017745)

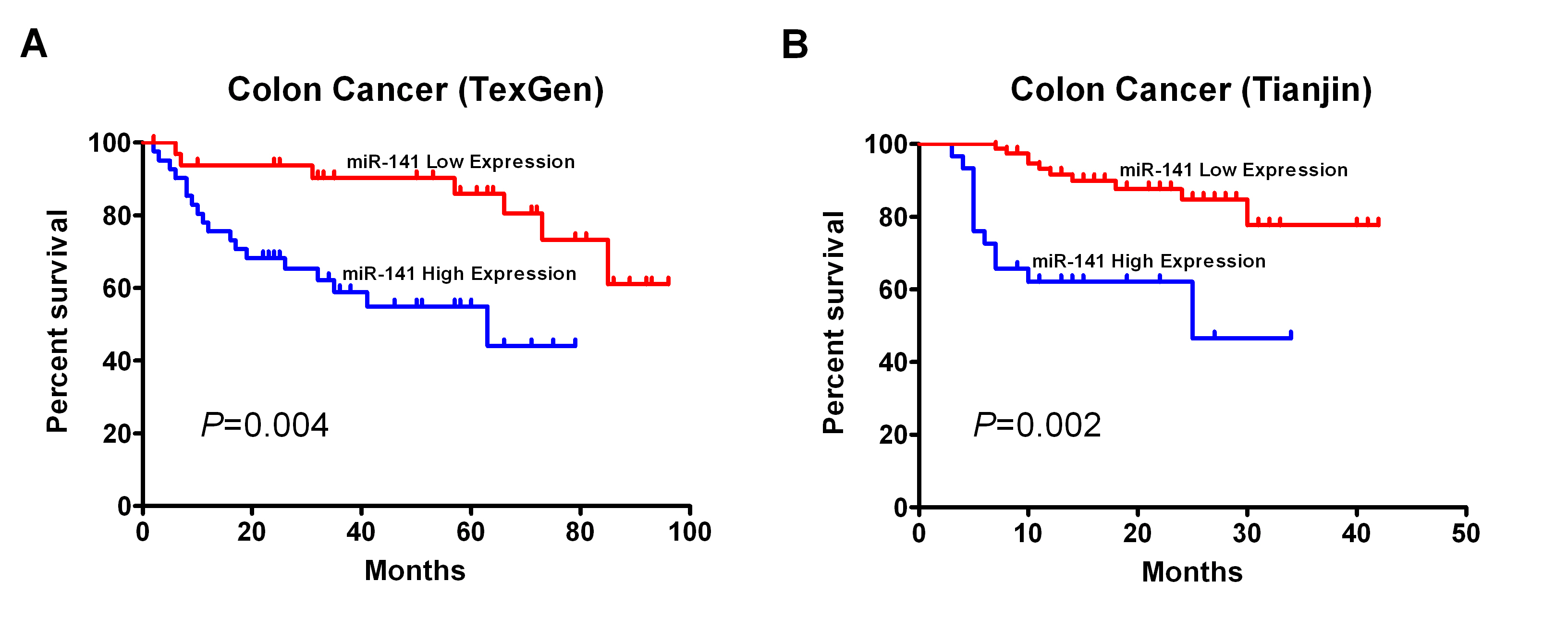

Supplement: Figure S1 — Higher miR-141 predicts poor prognosis in both cohorts. Kaplan-Meier survival curves for colon cancer patients in both cohorts. The survival data were compared using the log-rank test and miR-141 expression levels in patients defined as high or low relative to the median. P-value of log-rank test is 0.004 and 0.002 in TexGen (A) and Tianjin (B) cohorts, respectively. Higher plasma levels of miR-141 were associated with poor overall survival in colon cancer patients. (JPG) [file pone.0017745.s001.jpg]
