# Supplementary material for: Circulating Plasma MiR-141 Is a Novel Biomarker for Metastatic Colon Cancer and Predicts Poor Prognosis
Source: PLoS One. 2011 Mar 17;6(3):e17745. doi: 10.1371/journal.pone.0017745 (PMC3060165; doi:10.1371/journal.pone.0017745)
Supplement: Table S1 — (DOC) [file pone.0017745.s002.doc]

Table S1. Demographic information of normal controls and colon cancer patients in the training cohort from TexGen

| **Disease status** | **Gender** | **Race/Ethnicity** | **Average Age** |
| --- | --- | --- | --- |
| Normal n = 13 | F | W | 56.08 |
| Normal n = 1 | F | B | 53.00 |
| Normal n = 1 | F | H | 52.00 |
| Normal n = 10 | M | W | 55.60 |
| Normal n = 2 | M | A | 54.00 |
| Normal n = 1 | M | H | 50.00 |
| Stage I CRC n = 3 | F | W | 54.43 |
| Stage I CRC n = 5 | M | W | 54.22 |
| Stage II CRC n =10 | F | W | 53.80 |
| Stage II CRC n = 4 | M | W | 52.00 |
| Stage III CRC n = 9 | F | W | 52.33 |
| Stage III CRC n = 1 | F | B | 56.00 |
| Stage III CRC n = 1 | F | H | 60.00 |
| Stage III CRC n = 6 | M | W | 55.50 |
| Stage IV CRC n = 15 | F | W | 53.60 |
| Stage IV CRC n = 1 | F | A | 55.00 |
| Stage IV CRC n = 1 | F | H | 48.00 |
| Stage IV CRC n = 15 | M | W | 57.27 |
| Stage IV CRC n = 2 | M | B | 49.00 |
| Stage IV CRC n = 1 | M | H | 47.00 |

Race/Ethnicity: W (White), B (Black), and H (Hispanic)
