# Supplementary material for: Circulating Plasma MiR-141 Is a Novel Biomarker for Metastatic Colon Cancer and Predicts Poor Prognosis
Source: PLoS One. 2011 Mar 17;6(3):e17745. doi: 10.1371/journal.pone.0017745 (PMC3060165; doi:10.1371/journal.pone.0017745)
Supplement: Table S2 — (DOC) [file pone.0017745.s003.doc]

Table S2. Clinical characterization of CRC patient samples and normal controls in the validation set from Tianjin

| **Disease status** | **Gender** | **Race/Ethnicity** | **Average Age** |
| --- | --- | --- | --- |
| Normal n = 20 | F | Han | 61.10 |
| Normal n = 28 | M | Han | 60.29 |
| Stage I CRC n = 2 | F | Han | 47.50 |
| Stage I CRC n = 1 | M | Han | 63.00 |
| Stage II CRC n = 15 | F | Han | 56.07 |
| Stage II CRC n = 34 | M | Han | 64.21 |
| Stage III CRC n = 12 | F | Han | 59.92 |
| Stage III CRC n = 20 | M | Han | 60.75 |
| Stage IV CRC n = 15 | F | Han | 64.53 |
| Stage IV CRC n = 12 | M | Han | 64.33 |

Race/Ethnicity: Han Chinese constitute more than 90% of the population of China
